# Supplementary material for: Naturally Occurring Mutations within HBV Surface Promoter II Sequences Affect Transcription Activity, HBsAg and HBV DNA Levels in HBeAg-Positive Chronic Hepatitis B Patients
Source: Viruses. 2019 Jan 18;11(1):78. doi: 10.3390/v11010078 (PMC6356792; doi:10.3390/v11010078)
Supplement: Supplementary file 1 [file viruses-11-00078-s001.zip › viruses-419880-finalcheck-supplementary/20190113-Supplementary figures.doc]

**Figure S1.** The diagram of cases screening process. From 362 patients, 229 CHB patients infected with C-genotype HBV and their S gene were sequenced. According to relevant research reports and previous studies in this laboratory, point mutations in certain S genes may lead to elevated or decreased HBsAg values. Five point-mutations that increase HBsAg occur at amino acid positions sI68, sC76, sV177, sM198 and sI218 of the small S protein. The 13 point-mutations that cause a low detection of HBsAg are sE2, sL21, sR24, sT47, sC69*, sL95, sL98, sS117, sR122, sI126, sG145R, sW182*, and sV224. Excluding HBV S protein mutations in the above 18 mutations, the remaining serum samples of 113 patients were directly sequenced in the HBV SPII region PCR product, and finally successfully sequenced in 106 cases.

**Figure S2.** The difference value of mutation frequency between LS and HS group in every nucleotide site within HBV SPII. To screen the novel mutation within SPII, the difference value of mutation frequency was calculated (LS-HS) one nucleotide site by one. LS, low HBsAg; HS, high HBsAg.
