# Supplementary material for: Naturally Occurring Mutations within HBV Surface Promoter II Sequences Affect Transcription Activity, HBsAg and HBV DNA Levels in HBeAg-Positive Chronic Hepatitis B Patients
Source: Viruses. 2019 Jan 18;11(1):78. doi: 10.3390/v11010078 (PMC6356792; doi:10.3390/v11010078)
Supplement: Supplementary file 1 [file viruses-11-00078-s001.zip › viruses-419880-finalcheck-supplementary/20190113-Supplementary Tables.doc]

**Supplementary material**

Table S1. Primer list in this study.

| **Primer name** | **Direction** | **Sequence (5' to 3')** | **Purpose** |
| --- | --- | --- | --- |
| SPII (External) | F ^1^ | TCACATTCATTTACAGGAGGAC | SPII amplifying |
|  | R ^2^ | TGGGTGAGGCAGTAGTCG |  |
| SPII (Internal) | F | TTATGCCTGCTAGGTTCTA | SPII amplifying |
|  | R | GCTTGGTGGAATGTTGTG |  |
| pGL3-SPII | F | GGGGTACCTTCGGAGCCAACTCAAAC ^3^ | SPII cloning |
|  | R | GAAGATCTCCACTGCATGGCCTGAG ^4^ |  |
| pGL3-SPII-C18T | F | CCCCAACAAGGATCA***T***TGGCCAGAGGCAAAT ^5^ | pGL3-SPII-C18T cloning |
|  | R | ATTTGCCTCTGGCCA***A***TGATCCTTGTTGGGG |  |
| pGL3-SPII-C44T | F | CAAATCAGGTAGGAG***T***GGGAGCATTCGGGCC | pGL3-SPII-C44T cloning |
|  | R | GGCCCGAATGCTCCC***A***CTCCTACCTGATTTG |  |
| pGL3-SPII-A75T | F | AGGGTTCACCCCACC***T***CACGGCGGTCTTTTG | pGL3-SPII-A75T cloning |
|  | R | CAAAAGACCGCCGTG***A***GGTGGGGTGAACCCT |  |
| pGL3-SPII-G82A | F | TCACCCCACCACACGGC***A***GTCTTTTGGGGTGGAGC | pGL3-SPII-G82A cloning |
|  | R | GCTCCACCCCAAAAGAC***T***GCCGTGTGGTGGGGTGA |  |
| pGL3-SPII-A115C | F | GCCCTCAGGCTCAGGGC***C***TATTGACAACAGTGCCA | pGL3-SPII-A115C cloning |
|  | R | TGGCACTGTTGTCAATA***G***GCCCTGAGCCTGAGGGC |  |
| pGL3-SPII-T118C | F | CTCAGGCTCAGGGCATA***C***TGACAACAGTGCCAGTA | pGL3-SPII-T118C cloning |
|  | R | TACTGGCACTGTTGTCA***G***TATGCCCTGAGCCTGAG |  |
| pGL3-SPII-G120A | F | CAGGCTCAGGGCATATT***A***ACAACAGTGCCAGTAGC | pGL3-SPII-G120A cloning |
|  | R | GCTACTGGCACTGTTGT***T***AATATGCCCTGAGCCTG |  |
| pGL3-SPII-G129A | F | GGCATATTGACAACAGT***A***CCAGTAGCACCTCCTCC | pGL3-SPII-G129A cloning |
|  | R | GGAGGAGGTGCTACTGG***T***ACTGTTGTCAATATGCC |  |
| pGL3-SPII-T134C | F | ATTGACAACAGTGCCAG***C***AGCACCTCCTCCTGCCT | pGL3-SPII-T134C cloning |
|  | R | AGGCAGGAGGAGGTGCT***G***CTGGCACTGTTGTCAAT |  |
| pGL3-SPII-A138G | F | ACAACAGTGCCAGTAGC***G***CCTCCTCCTGCCTCCAC | pGL3-SPII-A138G cloning |
|  | R | GTGGAGGCAGGAGGAGG***C***GCTACTGGCACTGTTGT |  |
| pGL3-SPII-C189A | F | AGACAGCCTACTCCCAT***A***TCTCCACCTCTAAGAGA | pGL3-SPII-C189A cloning |
|  | R | TCTCTTAGAGGTGGAGA***T***ATGGGAGTAGGCTGTCT |  |
| pGL3-SPII-G228A | F | TCATCCTCAGGCCATGCA***A***CTCCACAACATTCCACCA | pGL3-SPII-G228A cloning |
|  | R | TGGTGGAATGTTGTGGAG***T***TGCATGGCCTGAGGATGA |  |
| pLMS/1.2/PC-C18T ^6^ | F | ACAAGGATCA***T***TGGCCAGAGGCAAATCAGGTAGGAG | pLMS/1.2/PC-C18T cloning |
|  | R | CTCTGGCCA***A***TGATCCTTGTTGGGGTTGAAGTCCC |  |
| pLMS/1.2/PC-G82A | F | ACCACACGGC***A***GTCTTTTGGGGTGGAGCCCTCAGG | pLMS/1.2/PC-G82A cloning |
|  | R | CCAAAAGAC***T***GCCGTGTGGTGGGGTGAACCCTGGC |  |
| pLMS/1.2/PC-A115C | F | GGCTCAGGGC***C***TATTGACAACAGTGCCAGTAGCAC | pLMS/1.2/PC-A115C cloning |
|  | R | TTGTCAATA***G***GCCCTGAGCCTGAGGGCTCCACCCC |  |
| pLMS/1.2/PC-G120A | F | AGGGCATATT***A***ACAACAGTGCCAGTAGCACCTCCTC | pLMS/1.2/PC-G120A cloning |
|  | R | CACTGTTGT***T***AATATGCCCTGAGCCTGAGGGCTCC |  |
| pLMS/1.2/PC-A138G | F | TGCCAGTAGC***G***CCTCCTCCTGCCTCCACCAATCGG | pLMS/1.2/PC-A138G cloning |
|  | R | AGGAGGAGG***C***GCTACTGGCACTGTTGTCAATATGC |  |
| pLMS/1.2/PC-C189A | F | CTACTCCCAT***A***TCTCCACCTCTAAGAGACAGTCATC | pLMS/1.2/PC-C189A cloning |
|  | R | GAGGTGGAGA***T***ATGGGAGTAGGCTGTCTTCCTGACTG |  |
| HBV total RNA | F | AAGCCACCCAAGGCACAG | Detect the level of HBV total RNA |
|  | R | GCACCAGCACCATGCAAC |  |
| HBV pgRNA | F | GAGTGTGGATTCGCACTCC | Detect the level of HBV pgRNA |
|  | R | GAGGCGAGGGAGTTCTTCT |  |
| RPS11 | F | CGTGACGAAGATGAAGATGC | Endogenous reference |
|  | R | GCACATTGAATCGCACAGTC |  |

^1^ F, forward primer；^2^ R, reverse primer；^3^ Underlined sequences, cleavage sites of KpnI; ^4^ Underlined sequences, cleavage sites of BglII; ^5^ bold and italic sequences, the position of single point mutation; ^6^ pLMS, pBluescript-LMS; pgRNA, pregenomic RNA; RPS11, Ribosomal protein S11; SPII, surface promoter II; 1.2/PC, pBlueBac4.5 1.2/PC.

Table S2. Comparisons of the main characteristics of the patients infected with C1- or C2- subgenotype.

| **Characteristics** | **C1 (n = 18)** | **C2 (n = 87)** | ***p*-value ^1^** |
| --- | --- | --- | --- |
| Demographic |  |  |  |
| Age, median (range) | 24.8±3.2 | 33.0±9.3 | 0.0003 |
| Gender M/F (% M) | 18/2 (90.0) | 63/24 (72.4) | 0.0998 |
|  |  |  |  |
| Virologic |  |  |  |
| HBV DNA (log IU/mL), median (range) | 7.9 (6.7-8.9) | 8.2 (6.6-9.8) | 0.1357 |
| HBsAg (log IU/mL), median (range) | 4.3 (3.5-5.2) | 4.4 (3.1-5.2) | 0.9019 |

^1^ For age, HBV DNA, and HBsAg, *p*-values represented the results of Mann-Whitney statistical tests between two groups. For gender, *p*-values represented the results of Chi-square statistical tests between two groups. M, male; F, female; HBsAg, Hepatitis B surface antigen.

Table S3. Comparisons of mutation frequencies in each SPII structural/functional regions between C1 and C2 sequences.

| **SPII regions** | **Position ^1^** | | **Mutation frequency (%)** | | ***p*-value ^2^** |
| --- | --- | --- | --- | --- | --- |
|  | **Starting** | **End** | **C1 (n = 18)** | **C2 (n = 87)** |  |
| A | 19 | 81 | 3.2 (36/1134) | 2.4 (134/5481) | 0.1790 |
| B | 84 | 103 | 0.0 (0/360) | 0.3 (6/1740) | 0.5982 |
| C | 104 | 140 | 4.7 (31/666) | 4.1 (132/3219) | 0.5241 |
| D | 141 | 160 | 0.3 (1/360) | 0.9 (15/1740) | 0.3352 |
| E | 164 | 185 | 1.0 (4/396) | 0.6 (12/1914) | 0.5000 |
| F | 176 | 200 | 0.9 (4/450) | 0.3 (6/2175) | 0.0758 |
| G | 200 | 224 | 1.3 (6/450) | 1.0 (22/2175) | 0.6116 |
| NF1 binding sites | 19 | 32 | 3.2 (8/252) | 2.4 (29/1218) | 0.5050 |
| Sp1 binding sites | 88 | 103 | 0.0 (0/288) | 0.3 (4/1392) | 1.0000 |
| Sp1 binding sites | 136 | 145 | 3.3 (6/188) | 3.9 (34/870) | 0.8333 |
| NF-γ binding sites | 150 | 169 | 0.3 (1/360) | 0.7 (13/1740) | 0.4870 |
| C/EBP binding sites | 150 | 169 | 0.3 (1/360) | 0.7 (13/1740) | 0.4870 |
| Sp1 binding sites | 176 | 185 | 0.6 (1/180) | 0.1 (1/870) | 0.3142 |
| Sp1 binding sites | 190 | 199 | 1.1 (2/180) | 0.2 (2/870) | 0.1381 |
| TCE binding sites | 209 | 216 | 0.0 (0/144) | 0.9 (6/696) | 0.5974 |
| CCAAT box | 155 | 159 | 0.0 (0/90) | 0.2 (1/435) | 1.0000 |
| Transcription initiation site | 208 | 208 | 0.0 (0/18) | 1.1 (1/87) | 1.0000 |

^1^ The numbering of HBV SPII nucleotide sequence referred to the position from consensus sequence. ^2^ *p*-values represented the results of Chi-square statistical tests. C/EBP, CCAAT/ enhancer binding protein; NF, nuclear factor; Sp, specificity protein; SPII, surface promoter II; TCE, transcriptional control element.

Table S4. Comparisons of the main characteristics between LS and HS patient groups with C2 infection.

| **Characteristics** | **LS ^1^ (n = 43)** | **HS (n = 44)** | ***p*-value ^2^** |
| --- | --- | --- | --- |
| Demographic |  |  |  |
| Age, median (range) | 33.3±9.2 | 32.7±9.6 | 0.7085 |
| Gender M/F (% M) | 29/14 (67.4) | 34/10 (77.3) | 0.3446 |
|  |  |  |  |
| Virologic |  |  |  |
| HBV DNA (log IU/mL), median (range) | 7.8 (6.6-9.1) | 8.5 (7.2-9.8) | 0.0000 |
| HBsAg (log IU/mL), median (range) | 4.0 (3.1-4.4) | 4.6 (4.4-5.2) | NA |
|  |  |  |  |
| Total mutation frequency in SPII (%) | 220/9804 (2.2) | 185/10032 (1.8) | 0.0465 |

**^1^** 87 patients were divided into low HBsAg group (LS) and high HBsAg group (HS) according to Median of HBsAg levels (23648.0 IU/ml). ^2^For age, HBV DNA, HBsAg and HBeAg, *p*-values represented the results of Mann-Whitney statistical tests between two groups; For gender and mutation detection rate, *p*-values represented the results of Chi-square statistical tests between two groups. M, male; F, female; HBsAg, Hepatitis B surface antigen; NA, not available.

Table S5. Comparisons of mutation frequencies in each SPII structural/functional regions between LS and HS groups with C2 infection.

|  | **SPII regions** | **Position ^1^** | | | **Mutation detection rates (%) ^2^** | | ***p*-value ^3^** |
| --- | --- | --- | --- | --- | --- | --- | --- |
|  |  | **Starting** | | **End** | **LS (n = 43)** | **HS (n = 44)** |  |
| A | | | 19 | 81 | 3.0 (80/2709) | 1.9 (54/2772) | 0.0178 |
| B | | | 84 | 103 | 0.6 (5/860) | 0.1 (1/880) | 0.1200 |
| C | | | 104 | 140 | 3.8 (61/1591) | 4.4 (71/1628) | 0.4781 |
| D | | | 141 | 160 | 0.8 (7/860) | 0.9 (8/880) | 1.0000 |
| E | | | 164 | 185 | 0.6 (6/946) | 0.6 (6/968) | 1.0000 |
| F | | | 176 | 200 | 0.4 (4/1075) | 0.2 (2/1100) | 0.4473 |
| G | | | 200 | 224 | 1.2 (13/1075) | 0.8 (9/1100) | 0.3982 |
| NF1 binding sites | | | 19 | 32 | 3.2 (19/602) | 1.6 (10/616) | 0.0922 |
| Sp1 binding sites | | | 88 | 103 | 0.4 (3/688) | 0.1 (1/704) | 0.3691 |
| Sp1 binding sites | | | 136 | 145 | 4.7 (20/430) | 3.2 (14/440) | 0.2963 |
| NF-γ binding sites | | | 150 | 169 | 0.6 (5/860) | 0.9 (8/880) | 0.5802 |
| C/EBP binding sites | | | 150 | 169 | 0.6 (5/860) | 0.9 (8/880) | 0.5802 |
| Sp1 binding sites | | | 176 | 185 | 0 (0/430) | 0.2 (1/440) | 1.0000 |
| Sp1 binding sites | | | 190 | 199 | 0.2 (1/430) | 0.2 (1/440) | 1.0000 |
| TCE binding sites | | | 209 | 216 | 0.9 (3/344) | 0.9 (3/352) | 1.0000 |
| CCAAT box | | | 155 | 159 | 0 (0/215) | 0.5 (1/220) | 1.0000 |
| Transcription initiation site | | | 208 | 208 | 2.3 (1/43) | 0 (0/44) | 0.4944 |

^1^ The numbering of HBV SPII nucleotide sequence referred to the position from consensus sequence. **^2^** 87 patients were divided into low HBsAg group (LS) and high HBsAg group (HS) according to Median of HBsAg levels (23648.0 IU/ml). ^3^ *p*-values represented the results of Chi-square statistical tests. C/EBP, CCAAT/enhancer binding protein; NF, nuclear factor; Sp, specificity protein; TCE, transcriptional control element.
